# Relationship between amino acid properties and functional parameters in olfactory receptors and discrimination of mutants with enhanced specificity

**DOI:** 10.1186/1471-2105-13-S7-S1

**Published:** 2012-05-08

**Authors:** M Michael Gromiha, K Harini, R Sowdhamini, Kazuhiko Fukui

**Affiliations:** 1 Department of Biotechnology, Indian Institute of Technology Madras, Chennai 600 036, Tamilnadu, India; 2 National Center for Biological Sciences, Bangalore, India; 3 Computational Biology Research Center, National Institute of Advanced Industrial Science and Technology, 2-4-7 Aomi, Koto-ku, Tokyo 135-0064, Japan

## Abstract

**Background:**

Olfactory receptors are key components in signal transduction. Mutations in olfactory receptors alter the odor response, which is a fundamental response of organisms to their immediate environment. Understanding the relationship between odorant response and mutations in olfactory receptors is an important problem in bioinformatics and computational biology. In this work, we have systematically analyzed the relationship between various physical, chemical, energetic and conformational properties of amino acid residues, and the change of odor response/compound's potency/half maximal effective concentration (EC50) due to amino acid substitutions.

**Results:**

We observed that both the characteristics of odorant molecule (ligand) and amino acid properties are important for odor response and EC50. Additional information on neighboring and surrounding residues of the mutants enhanced the correlation between amino acid properties and EC50. Further, amino acid properties have been combined systematically using multiple regression techniques and we obtained a correlation of 0.90-0.98 with odor response/EC50 of goldfish, mouse and human olfactory receptors. In addition, we have utilized machine learning methods to discriminate the mutants, which enhance or reduce EC50 values upon mutation and we obtained an accuracy of 93% and 79% for self-consistency and jack-knife tests, respectively.

**Conclusions:**

Our analysis provides deep insights for understanding the odor response of olfactory receptor mutants and the present method could be used for identifying the mutants with enhanced specificity.

## Background

Membrane proteins perform several functions, including the transport of ions and molecules across the membrane, binding to small molecules at the extracellular space, recognizing the immune system and energy transducers. Olfactory receptors (OR) are membrane proteins, belonging to the G Protein-Coupled Receptor superfamily, which are characterized by the presence of hydrophobic transmembrane domains. The odorant response of an organism by ORs to its environment forms the basis for our understanding in intra-species interactions, host-pathogen interactions, balance of chemicals, cell-cell interactions and other fundamental processes. It is evident that individual odorant can be recognized by multiple ORs and conversely, one type of OR can recognize multiple odorants with distinct binding affinities and specificities [1,2]. The binding and response of ORs with odorants are critical for the conversion of chemical information into electronic signals in olfactory sensory neurons [3,4]. Recent studies showed that mosquitoes' odorant receptors help the insects to find humans and, inadvertently, to transmit malaria [5,6]. Further, ORs have been analyzed to understand the mechanism of chloride uptake [7], modulation of signaling [8], functional architecture of olfactory system [9], unitary response [10], structural and functional plasticity at binding pocket [11] etc. Similar analysis has also been reported for identifying the binding site residues and binding specificity of protein-protein complexes [12-17].

The importance of specific amino acid residues in ORs and other membrane proteins has been demonstrated through site-directed mutagenesis experiments. The experimental data on EC50, maximal velocity of transport, odorant response, percentage uptake of compounds, affinity and specificity have been accumulated in the database for functional residues in membrane proteins [18]. Kuang et al. [19] measured the EC50 values for lysine in the wild type and mutants of 5.24 receptor. Luu et al. [20] elucidated the features of olfactory receptors for determining ligand specificity using different amino acid agonists. The structural basis for mouse OR to EC50 data has been analyzed by systematically substituting amino acid residues in different transmembrane helical segments [2,21]. Schmiedeberg et al. [22] carried out docking studies to understand the influence of different chemical compounds as well as due to mutations. On the other hand, computational methods have been proposed to understand the binding affinity of ligands with ORs using the template structure of rhodopsin [23-25].

In spite of these studies, the role of amino acid properties for the change of EC50 or odorant response has not yet been explored. Further, it is necessary to develop computational models to discriminate the mutants, which increase or decrease EC50. In this work, we have constructed different datasets of goldfish, mouse and human ORs for the mutants that change the odorant response, increase cAMP (adenosine 3'-5'-cyclic mono phosphate) and EC50 values. The differences in experimental data (EC50/odor response etc.) upon mutations have been related with physical, chemical, energetic and conformational properties of amino acid residues and the important amino acid properties have been brought out. The combinations of amino acid properties and the influence of neighboring and surrounding residues have been successfully used to relate the experimental functional data. Further, machine learning methods have been utilized to discriminate mutants with enhanced EC50 values.

## Materials and methods

### Datasets

We have developed a database, TMFunction, for functionally important amino acid residues in membrane proteins [18]. TMFunction has been searched for all functional data available for ORs. We obtained the experimental data, EC50, odorant response and cAMP increase for goldfish, mouse and human ORs. The final dataset contains 119 data with the following categories: (i) EC50: goldfish OR with Lys, 12; Arg : 12; Gly: 6 and Glu: 6; mouse OR: 28; Human OR: 7; (ii) odorant response: cAMP increase: 24 and Ca^2+ ^increase: 24.

### Amino acid properties

We used a set of 49 diverse amino acid properties (physical, chemical, energetic and conformational), which fall into various clusters analyzed by Tommi and Kanehisa [26] in the present study. The amino acid properties were normalized between 0 and 1 using the expression:

(1) $${\text{P}}_{\text{norm}}\left(\text{i}\right)=\left[\text{P}\left(\text{i}\right)-{\text{P}}_{\text{min}}]/[{\text{P}}_{\text{max}}-{\text{P}}_{\text{min}}\right],$$

where P(i), P_norm_(i) are, respectively, the original and normalized values of amino acid i for a particular property, and P_min _and P_max _are, respectively, the minimum and maximum values. Further, the numerical and normalized values for all the 49 properties used in this study along with their brief descriptions have been explained in our earlier articles [27,28] and are available on the web at http://www.cbrc.jp/~gromiha/fold_rate/property.html. These properties have been successfully used to understand the folding and stability of proteins [29-33].

### Computational procedure

The mutation induced changes in property values ΔP(i) was computed using the equation [29]:

(2) $$\Delta \text{P(i) = }{\text{P}}_{\text{mut}}\left(\text{i}\right)-{\text{P}}_{\text{wild}}\left(\text{i}\right)$$

where P_mut_(i) - P_wild_(i) are, respectively, the property value of the ith mutant and wild type residues, and i varies from 1 to N; total number of mutants. The computed difference in property values ΔP (X in Eqn. 3) was related with experimental EC50 or odorant response (Y in Eqn. 3) using single correlation coefficient (r). It is given by:

(3) $$\text{r}=[N\sum_{i=1}^{N}X_{i}Y_{i}-\text{(}\sum_{i=1}^{N}X_{i}\sum_{i=1}^{N}Y_{i}\text{)]/}\sqrt{\text{[}N\sum_{i=1}^{N}X_{i}^{2}-{\text{(}\sum_{i=1}^{N}X_{i})}^{2}\text{][}N\sum_{i=1}^{N}Y_{i}^{2}-{\text{(}\sum_{i=1}^{N}Y_{i})}^{2}\text{]}},$$

### Molecular modelling of mouse OR73

Mouse olfactory receptor 73 (OR73) sequence was obtained from NCBI (http://www.ncbi.nlm.nih.gov/guide/) using text search. The TM regions and topology (N-terminus OUT/IN) of the sequence were predicted using the transmembrane prediction server HMMTOP [34]. The sequences of OR73 and the template (bovine rhodopsin; PDB ID: 1F88A) were aligned using PRALINE-TM [35] server and manually edited using JALVIEW [36] Version 2.4. The TM region predictions from HMMTOP and the two conserved motifs MAYDRYVAIC and NPXXY in OR73 were used to guide and improve the alignment of the query and the template.

**Figure 1:**
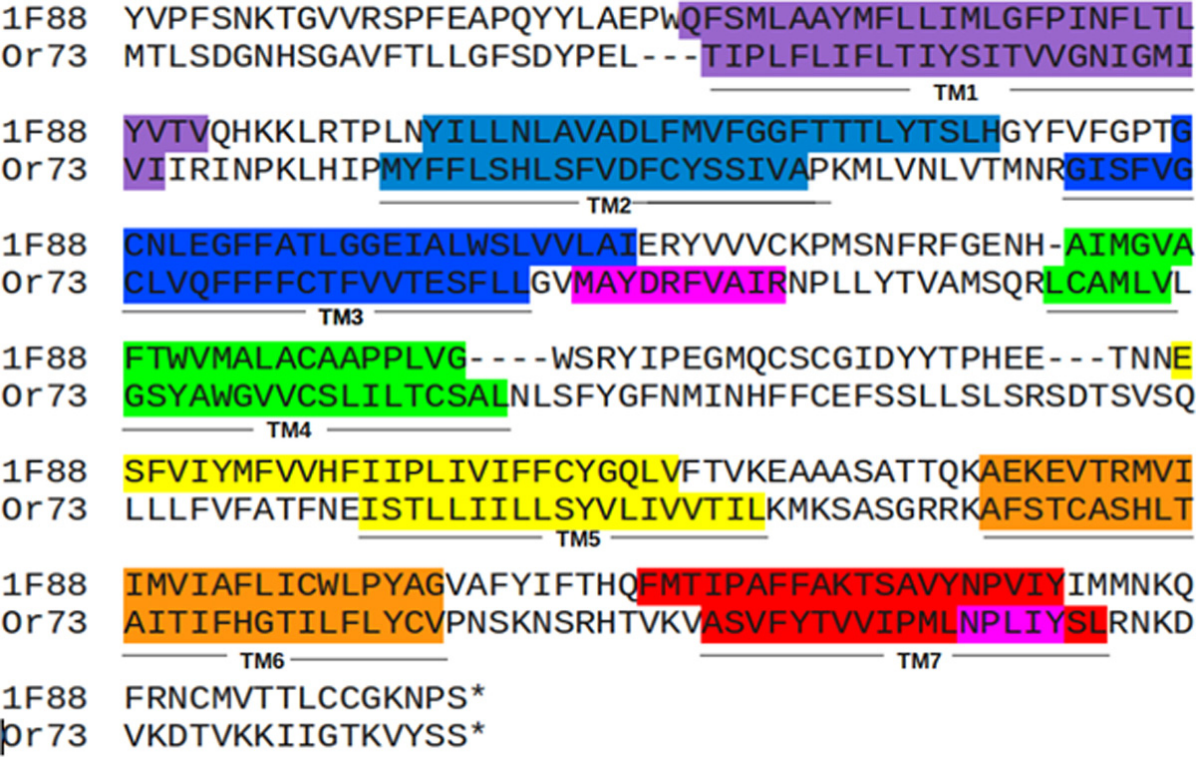
Alignment of query (OR73) with template (1F88). Observed and predicted TM helix positions are marked in VIGBYOR representation. Key functional motifs are shown in pink color against OR73 sequence.

The crystal structure of the bovine rhodopsin [23] (PDB ID: 1f88 chain A) was used as a template for the comparative modelling of the query (OR73). The structure of the template was obtained from RCSB (http://www.rcsb.org/pdb). The coordinates corresponding to the residues 236-239 and 328-333 were not available in the crystal structure of 1F88 chain A due to poor electron density and hence these residues were removed from the template sequence before the alignment. The final alignment (Figure 1) was used to construct the model using the software MODELLER [37] (version 9.8). A set of 20 structures were generated from which the five least probability density function models were validated by using PROCHECK [38] (Ramachandran Plot). The best structure was further energy minimized using the SYBYL software package (version 7.2) (Tripos Associates Inc.). Tripos force field, using 100 iterations of Powell's gradient with a distance dependent dielectric constant of 1 and a non bonded interaction cut off value of 8 and was terminated at a convergence of 0.05kcal mol. The final structures were further validated using PROCHECK [38].

### Local sequence and structural effects

The effect of local sequence, P_seq_(i), was included using the equation [29]:

(4) $$p_{\text{seq}}\left(\text{i}\right)=\left[\overset{j=i+k}{\underset{j=i-k}{\sum }}P_{\text{j}}\left(\text{i}\right)\right]-P_{\text{mut}}\left(\text{i}\right)$$

where, *P*_mut_(i) is the property value of the ith mutant residue and Σ*P*_j_(i) is the total property value of the segment of (2k+1) residues ranging from i-k to i+k about the i^th^ residue of wild type.

The structural information, *P*_str_(i), was included using the equation:

(5) $$P_{\text{str}}\left(\text{i}\right)=P_{\text{sur}}\left(\text{i}\right)-P_{\text{mut}}\left(\text{i}\right)$$

where *P*_mut_(i) is the property value of the i^th^ mutant residue, and:

(6) $$P_{\text{sur}}\left(\text{i}\right)=\underset{j}{\sum }{\text{n}}_{\text{ij}}\cdot P_{\text{j}}$$

where, n_ij _is the total number of type j residues surrounding the i^th^ residue of the protein within the volume of 8Å, and *P*_j_ is the property value of residue type j. Further details about the computation of surrounding residues have been described in our earlier articles [39,40].

### Multiple regression analysis

We have combined the amino acid properties using multiple regression technique: multiple correlation coefficients and regression equations were determined using standard procedures [41]. When fitting the data by multiple regression technique, reducing the number of variables increases the reliability of results. Hence, we selected three to five properties by searching all possible combinations of the 49 properties and computed the multiple correlation coefficients for all data sets. The highest correlation coefficient was selected and used in the analysis.

### Machine learning methods

We have analyzed several machine learning techniques implemented in WEKA program [42] for discriminating mutants with enhanced EC50 values. This program includes several methods based on Bayes function, Neural network, Radial basis function network, Logistic function, Support vector machine, Regression analysis, Nearest neighbor, Meta learning, Decision tree and Rules. The details of all these methods are available in our earlier articles [43-45].

### Jack-knife test

We have performed a jack-knife (leave-one-out cross-validation) test for assessing the validity of the present work. In this method, n-1 data are used for training and the left out mutant is used for testing.

### Assessment of predictive ability

We have used different measures, such as sensitivity, specificity and accuracy to assess the performance of discriminating mutants with enhanced function. These terms are defined as follows:

(7) $$\text{Sensitivity}=\text{TP}/\left(\text{TP}+\text{FN}\right)$$

(8) $$\text{Specificity}=\text{TN}/\left(\text{TN}+\text{FP}\right)$$

(9) $$\text{Accuracy}=\left(\text{TP}+\text{TN}\right)/\left(\text{TP}+\text{TN}+\text{FP}+\text{FN}\right),$$

where, TP, FP, TN and FN refer to the number of true positives, false positives, true negatives and false negatives, respectively.

## Results and discussion

### Relationship between amino acid properties and change in EC50 upon mutation: goldfish OR with Lys potency

**Figure 2:**
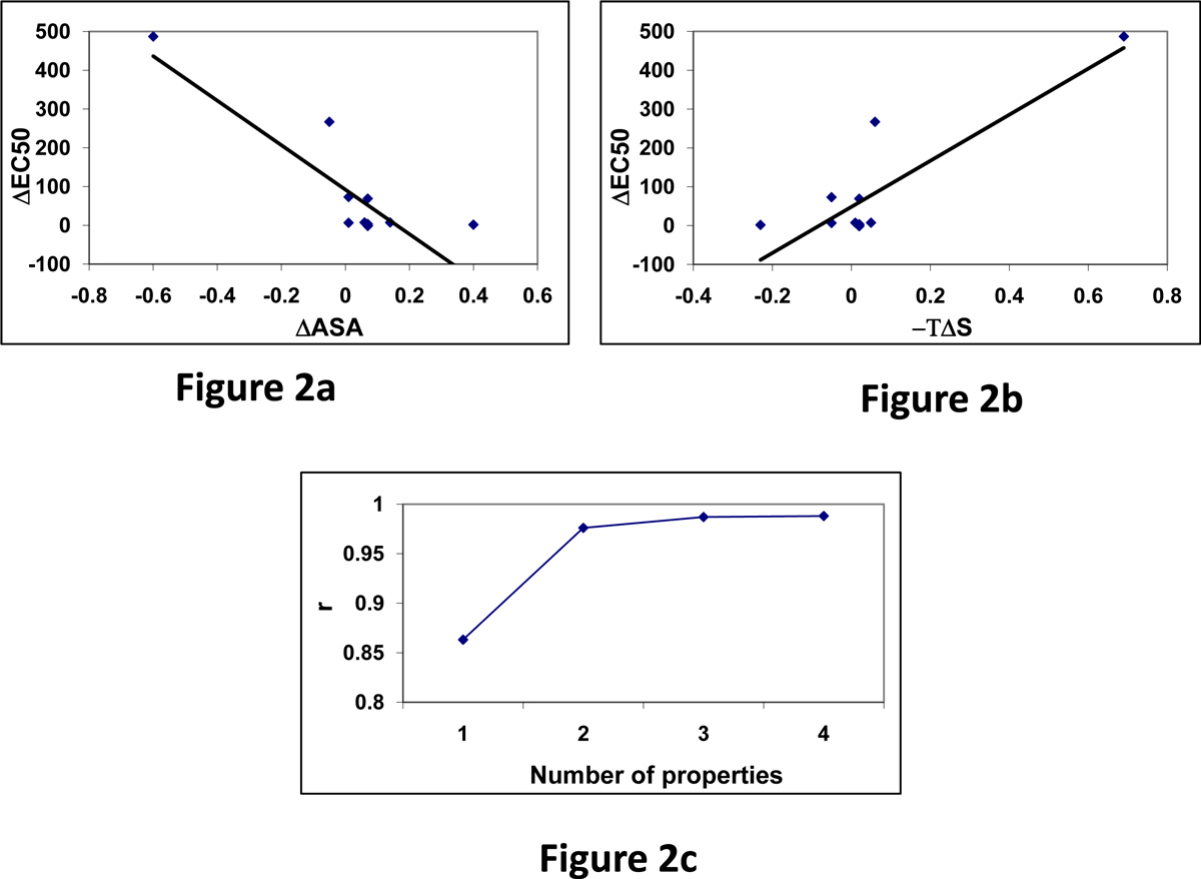
(a) Relationship between ΔASA and ΔEC50. (b) Variation of -TΔS with ΔEC50. (c) Variation of correlation coefficient with number of properties.

We have computed the changes in amino acid properties using Eqn.2 and related them with changes in EC50 upon mutations using Eqn. 3. The accessible surface area of unfolding showed a negative correlation of -0.86 for Lys potency with goldfish OR, and other physical properties, bulkiness, volume etc. showed appreciable negative correlation with ΔEC50 [46]. Figure 2a shows the relationship between ΔASA and ΔEC50. On the other hand, entropy change has high positive correlation with ΔEC50. Figure 2b shows the trend between -TΔS and ΔEC50 and the correlation coefficient is 0.85.

We have analyzed the combined effect of different amino acid properties and related with ΔEC50 values. The variation of correlation coefficient with number of properties is shown in Figure 2c. We noticed that the combination of four properties raised the correlation up to 0.988.

### Arg potency

**Figure 3:**
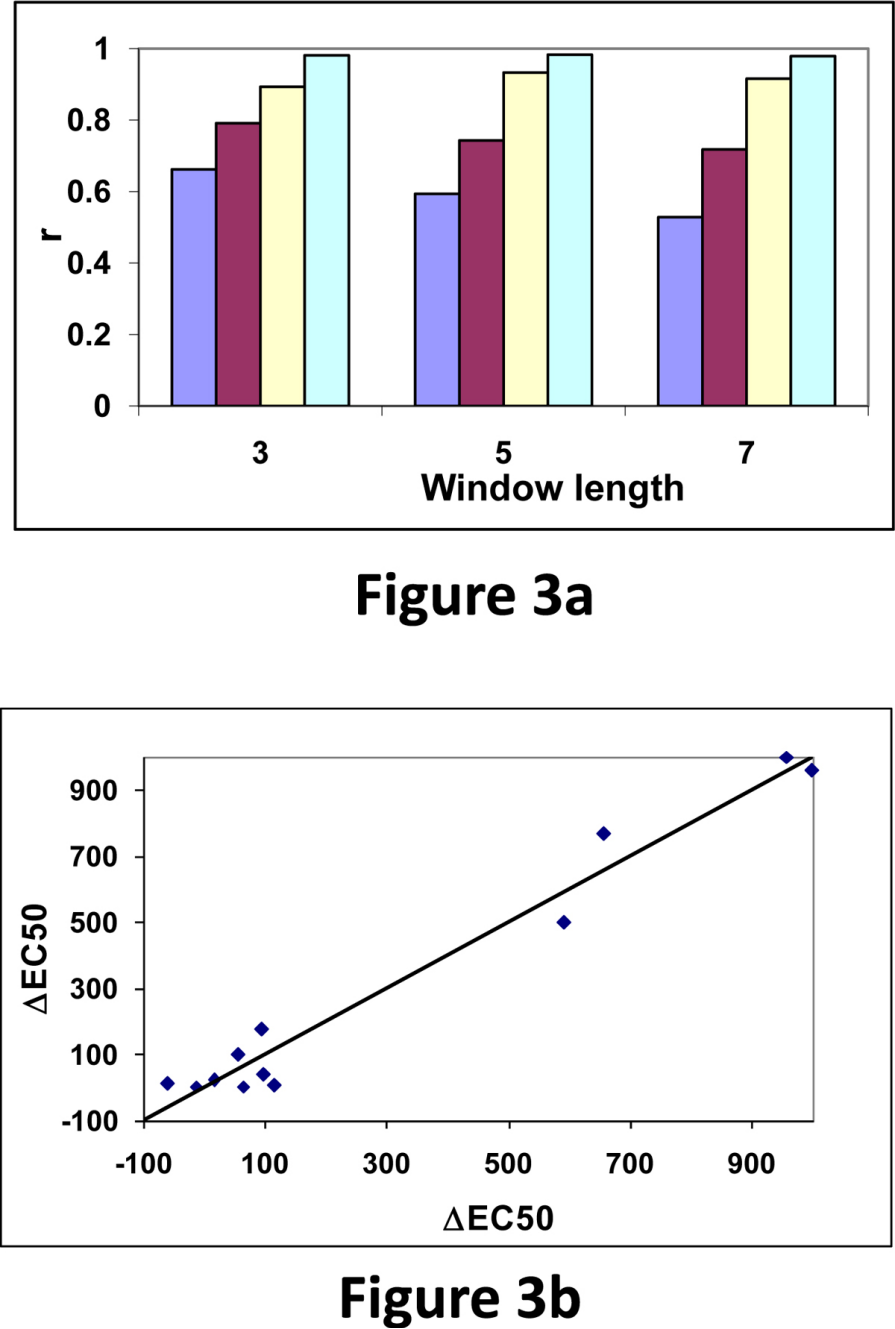
(a) Variation of correlation coefficients with different window lengths and combination of amino acid properties: purple: 1 property; brown: 2 properties; yellow: 3 properties and blue: 4 properties. (b) Relationship between experimental and predicted ΔEC50 values.

The data on Arg potency with goldfish OR showed a maximum correlation of 0.62 with the property, reduction in accessibility. Further, the combination of five properties also showed the maximum correlation of 0.90. Hence, we have included the information of neighboring residues along the sequence using Eqn. 4. We used different window lengths and the results for window lengths 3, 5 and 7 for the combination of 2, 3, and 4 amino acid properties is shown in Figure 3a.

We observed that the combination of four properties raised the correlation up to 0.98. This result shows that the experimental EC50 values are not depending on a specific residue and the information on neighboring residues are very important for the variation of EC50 upon mutation. The experimental and observed ΔEC50 values for all the 12 mutants are shown in Figure 3b and we noticed a good relationship between them. Further, the thermodynamic properties, ΔG, ΔH and -TΔS showed good correlation with ΔEC50 for goldfish OR with Glu and Gly potency.

### Molecular modeling and structural analysis of mouse OR

**Figure 4:**
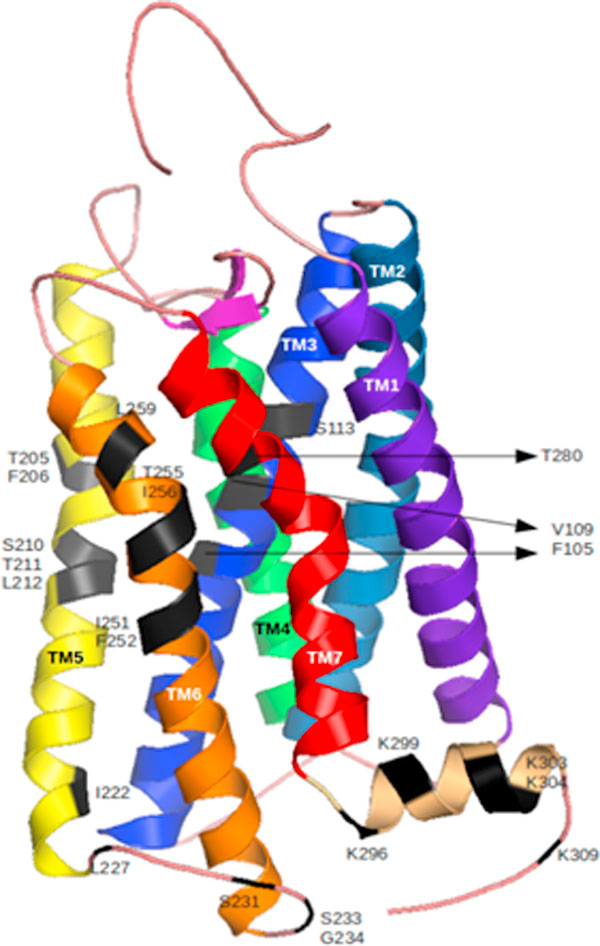
Homology model of mouse OR73 (7 TM helices-VIBGYOR, Mutations-Black).

A three-dimensional model of the mouse olfactory receptor, OR73, was obtained as mentioned in Methods. PROCHECK results for the model, excluding the loop regions, show more than 95% of residues in allowed regions (including strictly allowed and partially allowed) of the Ramachandran plot. The full-length structures of the models show more than 90% in the allowed regions (including strictly allowed and partially allowed) of the Ramachandran plot. The residues that were found in the disallowed regions were mainly in the loop regions which are highly variable in length and sequence identity. The RMSD of the full length OR model with respect to template was found to be less than 2Å .The amino acid mutations under study were marked on the model as shown in Figure 4. Many of the mutants lie in TM5 and TM6 and the loop in between has a glycine residue and this region could be implicated in the dimer interface. Most of the other 24 mutants are in intracellular loops.

The solvent accessibility of the amino acid residues, chosen for this study, were observed in the three-dimensional model. These values were calculated using PSA [47] within JOY version 3.2 [48,49] (Additional file 1). It is interesting that only 7 out of 24 mutants are solvent-buried and three of them are in TM3. Such solvent-buried residues could be important for the structural integrity of a protomer.

### Relationship between amino acid properties and change in EC50/cAMP increase/Ca^2+ ^increase upon mutation: mouse OR

Katada et al. [21] measured the EC50 values for the mutants at various positions in the transmembrane helices of mouse OR. Figure 4 shows a model for mouse OR and the information about mutated residues.

We have computed the difference in amino acid properties and related with difference in EC50 values. We observed a maximum correlation of just 0.38 and the combination of five properties raised the correlation only up to 0.56. We have included the information on neighboring residues, which increased the correlation up to 0.76. We then tried to utilize the structural information of mouse OR using Eqn. 5. The combination of mutants, neighboring residues and surrounding residues enhanced the correlation up to 0.81.

### cAMP level increase

Kato et al. [2] measured the increase in cAMP level and Ca^2+ ^for 24 mutants, which are located in transmembrane helical and loop regions. The analysis with overall data did not show high correlation and hence we classified the mutants based on their locations. Interestingly, the classification improved the correlation for the mutants both in transmembrane and loop regions.

**Figure 5:**
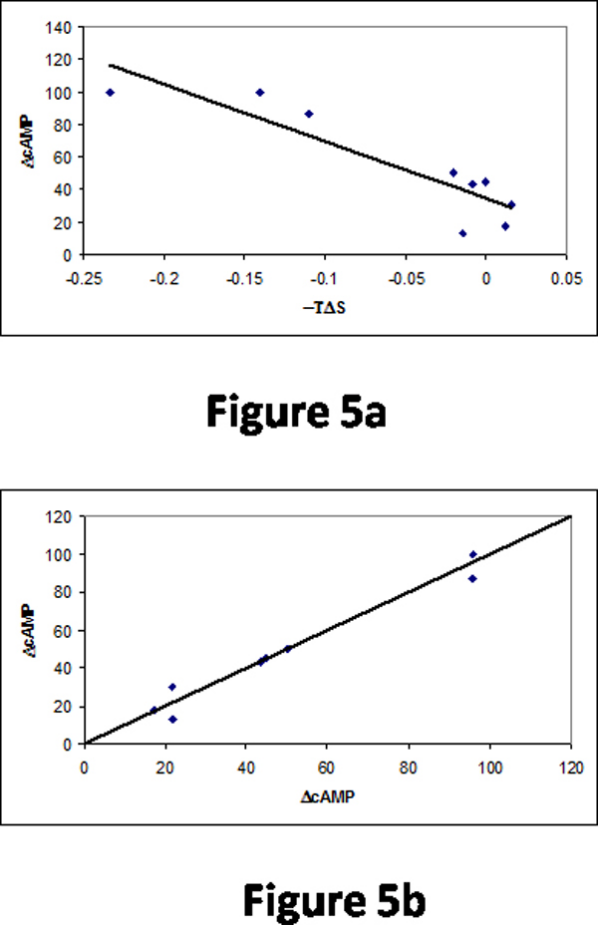
(a) Relationship between -TΔS and cAMP increase in the loop region of mouse OR. (b) Comparison of experimental and predicted cAMP increase levels with the combination of three properties.

We obtained the correlation of 0.86 with the combination of three properties for the mutants in membrane regions. In the loop region, a single property, -TΔS showed a correlation of -0.89 using the information of two neighboring residues on both sides of the mutants (Figure 5a). The combination of three properties raised the correlation up to 0.98. Figure 5b shows the relationship between the experimental and predicted cAMP level increase.

**Figure 6:**
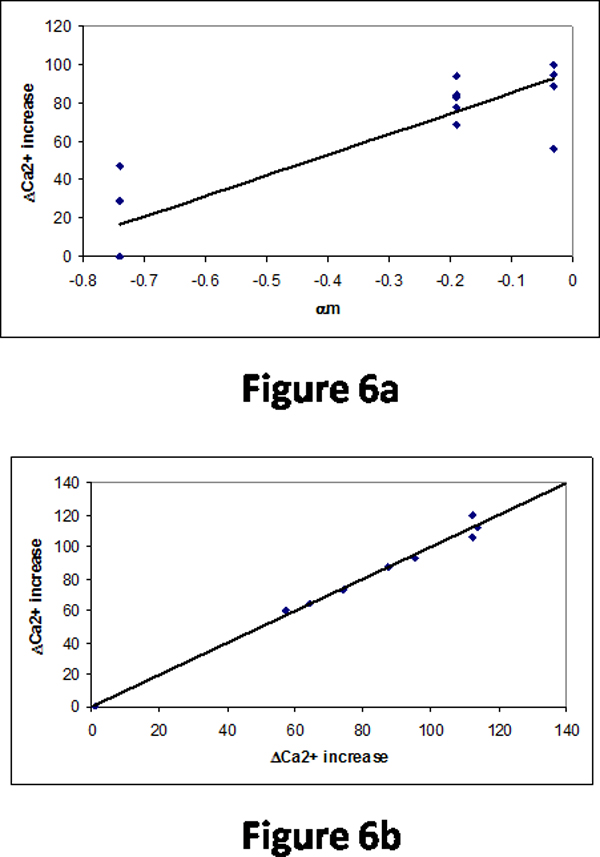
(a) Relationship between α_m _and Ca^2+ ^increase for the mutants in membrane spanning helical segments. (b) Comparison of experimental and predicted Ca^2+ ^increase for the mutants in loop regions.

We have analyzed the variation of Ca^2+ ^increase upon mutations and we observed that the classification of mutants based on their location is necessary for understanding the properties influencing Ca^2+ ^increase. The results obtained for transmembrane helical and loop regions are shown in Figures 6a and 6b, respectively. We obtained a maximum single correlation of 0.90 with the propensity of residues in the middle of α-helix for the mutants in transmembrane helices (Figure 6a). The relationship between experimental and predicted Ca^2+ ^increase for the mutants in the loop regions using a combination of three properties is shown in Figure 6b. The correlation coefficient is 0.995.

### Relationship between amino acid properties and change in EC50 upon mutation: human OR

**Figure 7:**
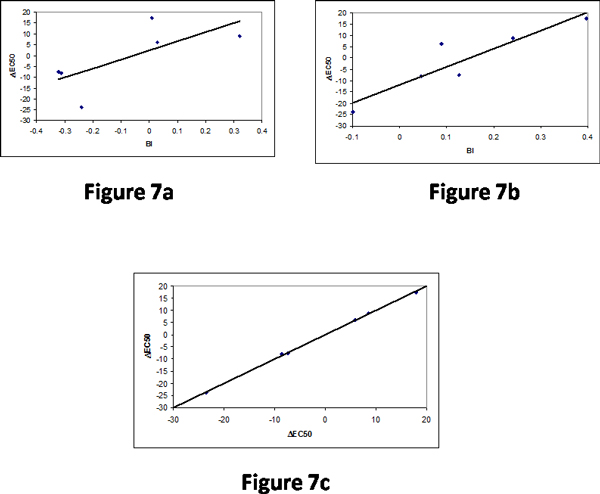
Relationship between bulkiness and ΔEC50 in human OR; (a) Mutant information and (b) Three neighboring residues (c) Comparison of experimental and predicted ΔEC50.

Schmiedeberg et al. (2007) measured the EC50 values for seven mutants in human OR. We found that bulkiness has a correlation of 0.71 and the information on three neighboring residues increased the correlation up to 0.92 (Figures 7a and 7b). This analysis reveals the importance of neighboring residues for determining the change in EC50. Further, the combination of just two properties increased the correlation up to 0.999 and Figure 7c showed the relationship between experimental and predicted ΔEC50.

### Discrimination of mutants that enhanced/decreased EC50: mouse OR

We have collected a set of 28 data in mouse OR in which 15 of them increased EC50 upon mutation and 13 mutants decreased the EC50 values. We made an attempt to discriminate these mutants using the information on wild type residue, mutant residue and the properties of neighboring residues. We have utilized several machine learning techniques for discrimination. Our method showed an accuracy of 92.9% for self-consistency test and the sensitivity and specificity are 93.3% and 92.3%, respectively. The assessment using jack-knife test showed an accuracy of 78.6% and the sensitivity and specificity are 80.0% and 76.9%, respectively. This method can be used to identify the mutants with increased/decreased EC50.

## Conclusions

We have constructed different datasets for mouse, goldfish and human ORs and various experimental data such as EC50, odorant response, Ca^2+ ^increase etc. The experimental data have been systematically analyzed with physical, chemical, energetic and conformational properties of amino acid residues and important properties have been listed out. We found that the information on neighboring and surrounding residues, namely the inclusion of motifs, is important to understand the function. Further, we have combined the amino acid properties using multiple regression analysis, which relates experimental EC50/cAMP level increase and Ca^2+ ^increase very well. We have utilized machine learning techniques for discriminating the mutants that enhance/reduce IC50 upon mutation and we obtained an accuracy of 93% and 79%, respectively, for self-consistency and jack-knife tests. The results obtained in the present work would help to understand the importance of amino acid properties to the functions of ORs and to identify the mutants with enhanced EC50 values.

## Competing interests

The authors declare that they have no competing interests.

## Authors' contributions

MMG, RS and KF conceived the project. MMG carried out the computations and wrote the manuscript. KH modeled the structures of ORs and analyzed them. MMG, KH, RS and FK contributed in discussions. KH and RS wrote the sections about modeling. All authors read and finalized the manuscript.

## Supplementary Material

Additional file 1 **Solvent accessibility of amino acids under study**. Click here for file
