# Supplementary material for: Relationship between amino acid properties and functional parameters in olfactory receptors and discrimination of mutants with enhanced specificity
Source: BMC Bioinformatics. 2012 May 8;13(Suppl 7):S1. doi: 10.1186/1471-2105-13-S7-S1 (PMC3348020; doi:10.1186/1471-2105-13-S7-S1)
Supplement: Additional file 1 — Solvent accessibility of amino acids under study. [file 1471-2105-13-S7-S1-S1.doc]

**Additional file 1: Solvent accessibility of amino acid residues under study**

| Amino acid | Position | Solvent | Accessibility* |
| --- | --- | --- | --- |
| residue | in the model | Model before minimisation | Energy minimized model |
| Location-TM helices |  |  |  |
| F105 | TM3 | 4.2 | 0 |
| V109 | TM3 | 22.2 | 1.4 |
| S113 | TM3 | 14.5 | 5.8 |
| T205 | TM5 | 47.4 | 47.9 |
| F206 | TM5 | 62.7 | 59 |
| S210 | TM5 | 66.7 | 58.2 |
| T211 | TM5 | 5.1 | 1.3 |
| L212 | TM5 | 39.6 | 32.8 |
| I251 | TM6 | 80.2 | 86.6 |
| F252 | TM6 | 42.2 | 35.2 |
| T255 | TM6 | 61.6 | 64 |
| I256 | TM6 | 10.1 | 7.4 |
| L259 | TM6 | 60.8 | 65.5 |
| T280 | TM6 | 1.2 | 0 |
| I222 | TM5 | 4.6 | 2.4 |
| Location-Intracelluar loops |  |  |  |
| K296 | c-ter | 49.2 | 42.6 |
| K299 | c-ter | 37.5 | 45.7 |
| K303 | c-ter | 44.1 | 46.8 |
| K304 | c-ter | 25.5 | 26.3 |
| K309 | c-ter | 60 | 57 |
| G234 | IC3 | 81.3 | 70.7 |
| S233 | IC3 | 26 | 31.1 |
| S231 | IC3 | 50.1 | 45 |
| L227 | IC3 | 77.8 | 79.2 |

(* Accessibility: <25%: buried, 25-50%: partially buried, >50%: solvent exposed)
